# Understanding Violence against Women Irregular Migrants Who Arrive in Spain in Small Boats

**DOI:** 10.3390/healthcare8030299

**Published:** 2020-08-26

**Authors:** María del Mar Jiménez-Lasserrotte, Esperanza López-Domene, José Manuel Hernández-Padilla, Cayetano Fernández-Sola, Isabel María Fernández-Medina, Karim El Marbouhe El Faqyr, Iria Dobarrio-Sanz, José Granero-Molina

**Affiliations:** 1 Spanish Red Cross, 04002 Almería, Spain; esperanzalopezdomene@hotmail.es; 2 Nursing, Physiotherapy and Medicine Department, Faculty of Health Sciences, Universidad de Almeria, 04007 Almería, Spain; j.hernandez-padilla@ual.es (J.M.H.-P.); cfernan@ual.es (C.F.-S.); Isabel_medina@ual.es (I.M.F.-M.); abogadokmf@gmail.com (K.E.M.E.F.); ids135@ual.es (I.D.-S.); jgranero@ual.es (J.G.-M.); 3 Adult, Child and Midwifery Department, School of Health and Education, Middlesex University, London NW4 4BT, UK; 4 Associate Researcher, Faculty of Health Sciences, Universidad Autónoma de Chile, Temuco 4780000, Chile

**Keywords:** sexual violence, abuse, sexual exploitation, public health, women irregular migrants, qualitative

## Abstract

African irregular migrants risk their lives crossing the Mediterranean Sea in small boats hoping to reach Europe. Women irregular migrants (WIMs) are an especially vulnerable group that suffer from violence and sexual aggression, but little is known about their actual experiences. The objective of our study is to describe and understand the violence against WIMs who arrive in Spain in small boats. A qualitative study based on Gadamer’s phenomenology was used. The data collection included twenty-six in-depth interviews with WIMs. Three main themes arose: “Poverty and discrimination push WIMs into migrating”; “WIMs as a paradigm of extreme vulnerability”, and “WIMs in small boats should raise the alarm”. WIMs who arrive to Europe in small boats have a history of violence, rape, prostitution, forced pregnancy, and human trafficking. Emergency care must include gynecological examinations and must make detecting sexual violence and human trafficking of WIMs part of their care protocols.

## 1. Introduction

Migrant and refugee movement is considered to be a political, social, and public health challenge around the world [1]. The European Union (EU) receives thousands of migrants and refugees annually, they are fleeing from war [2,3], sexual violence [4], racial, political, or religious conflicts, or extreme poverty [5,6]. Irregular migrants (IMs) are not authorized to enter or stay in the country to which they migrate, as they do not have legal permission, documentation, or refugee status. Usually, IMs come to the EU from the Middle East, Maghreb, and Sub-Saharan Africa [7]. Despite strict border control, in 2015 and 2016, 1,200,000 migrants crossed the Mediterranean into Europe and at least 6000 went missing at sea [8,9]. Many of them arrived in small boats to the coasts of Italy, Greece, or Spain. In 2017, 1200 small boats reached Spain, with a total of 22,419 migrants and a confirmed death toll of 249 [10]. The demographic profile of IMs has varied in the last few years, and the number of women and children has increased [11]. Women irregular migrants (WIMs) are especially vulnerable to violence throughout all of the migratory stages [12], therefore, understanding their experiences may aid healthcare providers in their treatment and care.

The fight against unwanted sea migration in Southern Europe has transformed borders as we know them, as well as the relationship between countries [13,14]. Despite border protection on the outer borders of the EU [15], irregular migration has seen a major increase since 2009, thousands of IMs try to get to Spain through the Mediterranean Sea in small boats [16]. After a dangerous journey from their countries of origin, IMs embark on small boats in northern Morocco towards the EU [7]. Controlled by trafficking networks and surrounded by violence, IMs risk their lives on the high seas [17]. Nongovernmental organizations such as Doctors Without Borders or the Red Cross [8,18] have taken on the responsibility of rescuing and providing emergency care to IMs [11]. In Spain, Maritime Rescue Team intercept the small boats offshore and alert the Red Cross Emergency Response Team (doctor, nurses, emergency medical technicians, and cultural mediators). The emergency care provided to IMs includes first aid care, triage, and treating hunger, dehydration, hypothermia, injuries, and psychosocial support [5]. The arrival in the EU of WIMs in small boats poses health, humanitarian, and social risks that can have an impact on an epidemiological [19], political [20], and healthcare provision level [21]. WIMs are more vulnerable as, in addition to physical or psychological problems, they are more exposed to violence [2], sexual exploitation, unwanted pregnancy [3,22,23], or genital mutilation [24].

WIMs may suffer from violence throughout all the stages of the migratory process, from recruitment, transit, and finally, during integration in the place they land [18]. While epidemiological studies [25], demographic studies [11], cultural studies [26], or gender violence studies are available on this topic [27,28], little is known about the experiences of WIMs who arrive in the EU in small boats. Understanding the perspective of WIMs would enable healthcare providers to better comprehend their experiences, better meet their needs, and improve first emergency care [29]. The framework developed by Zimmerman et al. [30], which divides the migratory process into the stages of recruitment, transit, exploitation, and integration/reintegration, allows us to study the experiences of WIMs. The objective of our study is to describe and understand the violence against WIMs who arrive in Spain in small boats.

## 2. Materials and Methods

### 2.1. Study Design and Setting

A qualitative design based on Gadamer’s hermeneutic phenomenology was used in the data interpretation and analysis [31]. Hermeneutic phenomenology describes how phenomena are experienced by individuals, centered around their meaning. Understanding the meaning that people assign to their experiences provides the theoretical base which supports practice. To Gadamer, understanding a text/transcription involves addressing prejudice, culture, tradition, and language. The study took place in the Spanish Red Cross’ facilities.

### 2.2. Participants

A purposive sample was used to recruit WIMs. The inclusion criteria for the WIMs was to have crossed the Mediterranean Sea in a small boat in the last 5 years. The exclusion criteria were to refuse to participate in the study and to be <18 years old.

### 2.3. Data Generation

In-depth interviews were carried out with twenty-six WIMs, with an average duration of thirty four minutes. In-depth interviews (Dis) were completed between February 2017 and April 2018 (Table 1). The DIs with WIMs began with the question “What was your experience regarding violence on the migratory journey?” The final question was, “Is there anything else you would like to add to the topic?” The DIs were carried out by two researchers who had received training and practiced the protocol. An open-ended question script was used (Table 2). All participants’ responses were recorded, transcribed, and incorporated into a hermeneutic unit that was later analyzed using the software Atlas-ti 8.0. Upon reaching data saturation, data collection ceased.

### 2.4. Data Analysis

A modified version of the stages developed by Valerie Fleming was used [32]. In the first step, the relevance of the research question was assessed; the researchers responded affirmatively to the question, “Can one study the violence against WIMs who arrive in Spain in small boats from a phenomenological perspective?” The second step was a reflection about the phenomenon. The third step aimed to achieve an in-depth understanding through dialogue with the participants. New questions emerged such as “What situations of violence affect WIMs while they wait to embark on small boats?” The understanding of the phenomenon through a dialogue with the text took place in the fourth step. Examining each sentence allowed themes, subthemes, and units of meaning to be identified. After reading the transcriptions, new questions emerged such as “How do being pregnant or traveling with children affect the violence against WIMs?” The trustworthiness and credibility of the results in the context of the study were established in the fifth step, guidelines established by the Consolidated Criteria for Reporting Qualitative Research were followed [33]: (a) cross-triangulation by three members of the research team, (b) auditing of the materials obtained from five randomly selected cases by an external independent researcher (revision de data collection and analysis), (c) post-interview (transcripts returned) and verification by participants post-analysis (participant checking).

### 2.5. Ethical Considerations

Approval was gained from the Spanish Red Cross Ethics and Research Committee (protocol number: CR-1501). The participants were informed of the purpose of the study. Permission was requested to record the conversations, participants signed an informed consent form, and their anonymity has been maintained at all times.

## 3. Results

Twenty-six WIMs, originating from eight African countries, participated in the study. Their average age was 28.8 years old (SD = 6.09). Of the WIMs, 42.3% traveled alone, 23.07% while pregnant, 23.07% with children, 7.7% while pregnant and with children, and 3.85% with siblings. Participants’ individual demographic characteristics are presented in Table 1. Our results have been structured into units of meaning, subthemes, and themes (Table 3).

### 3.1. Poverty and Discrimination Push WIMs into Migrating

This theme describes the motives behind taking on an extremely risky journey for WIMs. In migrants’ countries of origin, a culture of emigration as a means to escape discrimination and improve living conditions has arisen in recent years. The media offer images of a European paradise, which pushes women to risk their lives on the journey to escape from war, poverty, violence, or discrimination.

#### 3.1.1. The Difficulty of Living in Social and Economic Inequality

The majority of the women are from countries that have economic and social inequality with few resources for survival. This situation makes accessing education and escaping poverty or social exclusion nearly impossible. In addition to violence and gender discrimination, imagining an idealized life in Europe with equal opportunities drives them to go on a high-risk migration journey.

“I want to come to Europe so we can all live a better life, because women are equal to men here.”

(WIM-6)

The WIMs we interviewed lacked prospects for the future in their countries of origin and coming to Europe seemed like the only option to improve their living situation. Sometimes, the loss of a male figure leaves the family unprotected, in social and economic vulnerability, women are responsible for their families, which they pay for with their freedom. As one WIM comments, this can be the trigger to finally make the journey.

“My father died, and he had a debt that we could not pay. So it would be forgiven, I was forced to get married against my will at 16 years old. My mother begged me for forgiveness every day, ... but she also begged me not to run away, because (husband’s name) could retaliate against the whole family.”

(WIM-11)

#### 3.1.2. Escaping a Culture of Discrimination against Women

Along with arranged or forced marriage, sexual and physical abuse and violence may be present even within their own families. Many WIMs come from countries where they suffer genital mutilation as young girls as a way of following tradition and religion or controlling their sexuality. As some of our participants say, they flee from these practices.

“My first daughter died because of genital mutilation. I refused to continue that practice and I was publicly punished by my husband, closed in a room for a week. People assaulted me and gave me food through a window.”

(WIM-24)

WIMs flee from practices and customs focused on the control and reinforcement of the inferiority of women. In their cultures and countries of origin, domestic violence usually comes from someone close to them, that they know well. As one WIM says, some have been hit by their parents or husbands.

“I was with my partner for 4 years, and I suffered from physical and psychological abuse. My ex mother-in-law didn’t help me, ... she said it was normal, that all men did it.”

(WIM-12)

There are certain ethnicities with deep-rooted traditions that violate women’s rights. As one participant, who is of the “Tsimni” race, informed us, when a woman and a man are married, if he dies, she must marry one of his brothers to ensure the continuation of the family lineage. If she refuses, her life may be at risk:

“Four months after my husband died, I refused to get married again, and I was harassed and abused by my husband’s family... I had no choice but to flee!”

(WIM-14)

#### 3.1.3. Recruiting WIMs in Their Countries of Origin

On some occasions, people with power in their communities of origin, or even families of the victims themselves, contribute to the process of recruitment. There is a type of social coverage that protects these practices, and sending a daughter to a foreign country can imply gaining a position of higher status within the community. As one WIM says, the family often actively participates in financing and organizing at least one part of the dangerous journey:

“I asked my grandfather’s brother for money, I spoke with him and he organized it, … then I worked to repay him the money he had lent me.”

(WIM-3)

On other occasions, it is the trafficking network that recruits the victims directly, scamming them by promising them a bright future in Europe with a steady job as a hairdresser or taking care of children. In some communities, the agreement with the network of traffickers is sealed by a ceremony (juju or voodoo), which exercises control over the women. Juju is sometimes used to enforce a contract or ensure compliance. Coerced by magic rituals (including animal sacrifice) they believe that if they do not obey the trafficking network, their family will be in danger. As one participant explained:

“A Nigerian woman (madame) told me, ‘You are a very pretty woman and I can help you find work in Europe,’ She took care of my passport and the trip. She also made me do voodoo rituals: she took a blood sample from me, my hair, pubic hair and fingernails, and made me drink it. She said it would protect me, but if I didn’t obey, the spirits that were summoned would turn against my family and me.”

(WIM-16)

The trafficking network organizes the trip and the economic conditions. One man, from Morocco or Algeria, manages the payment with a friend or a relative of the woman. With the collusion of their families, these women often pawn the few personal or family belongings they have in order to pay for the journey. WIMs are excluded from taking part in these decisions:

“I didn’t know anything, nobody informed me, my brother paid a friend of Abiyán, who spoke with someone from Morocco who managed all the other things.”

(WIM-6)

In addition, the human trafficking ring can act directly by kidnapping these women. WIMs can be tricked, kidnapped, forced to hide to avoid being discovered, transported secretly, and sexually exploited. As one participant told us:

*“When I paid him* (the trafficking network)*, he told me: “We’ll pick you up in a parking lot”. When I got in the car, I saw that there were two other girls in the trunk.”*

(WIM-8)

### 3.2. WIMs as a Paradigm of Extreme Vulnerability

During the migratory journey from sub-Saharan countries to the Maghreb, WIMs are exposed to xenophobia, physical violence, and sexual exploitation. Consequently, as they wait to embark towards Spain, WIMs stay hidden in areas near the coast of Morocco or Algeria, where they survive in precarious conditions, trying to avoid being detained or deported.

#### 3.2.1. Sexual and Reproductive Control: The Journey Husband

Once the conditions are negotiated, WIMs are moved throughout different countries using the routes with the laxest migratory controls. The traffickers know how and where to go to avoid hitting border control, although there are always cities where they have to stop; these are known as “hot spots”. The journey is done on foot, or by bus, truck, taxi, or private cars. As one WIM says, threats are used as a way to control women throughout the trip:

“From Senegal to Morocco, they were constantly asking us for money and they threatened to make me go back. They made us dress in Arabic clothing, they made me wear a hijab, we always traveled hidden among suitcases.”

(WIM-9)

The women do not travel alone and are always accompanied by a woman or man who is in charge of their transportation, managing the payments and controlling the victims. Border crossing is one of the most traumatic moments for WIMs because the trafficking network may use them as bartering currency to bribe the police. In Morocco, WIMs stay half-hidden for long periods in flats in unsanitary conditions or in the forest (near the Spanish border) waiting to cross the Mediterranean in small boats. As one WIM says, they are obligated to beg or are exploited as domestic servants or prostitutes:

“I stayed hidden with my son in the forest outside Nador for three and a half months until we could leave on a small boat. It was so hard! I had to beg for food just to eat!”

(WIM-12)

At this point, WIMs already belong to a human trafficking network that makes all the decisions for them, including their sexual and reproductive health. They decide where the WIM will be prostituted and if she will use any contraceptive methods. If the WIM is pregnant, the human trafficking network will decide whether she will abort or carry the baby to term. As one WIM tells us, the trafficking network designates women to be in charge of controlling WIMs:

“Yes, a woman is in charge, she threatens you, ... you have to obey. I was abused! I had to pay back my stay.”

(WIM-18)

In the case that the pregnancy comes to full term, the baby would become property of the trafficking network. These children are usually the result of rapes and sexual abuse and usually go on the journey by small boat with the mother.

“My husband stayed in the forest, he said for me to go first because I am pregnant and that I should have the baby in Europe. I don’t know when he’ll get here, he hasn’t said anything!”

(WIM-5)

#### 3.2.2. Waiting to Board: Between Worker Exploitation and Sexual Slavery

Before leaving for Spain, WIMs survive in wooded areas in improvised camps made up of shelters built from branches and plastic tarps where they stay hidden for months on end. In these places, women suffer from racial discrimination, sexual harassment, and rape at the hands of the local population. As one WIM describes:

“I was so hungry, I went weeks without eating, sleeping in the streets! They treated us like animals, I don’t want to remember the time I spent there!”

(WIM-20)

The wait becomes unbearable, WIMs remain detained, isolated, and without any chance of getting help. These camps are places where fear and anxiety run deep and WIMs are controlled by men who do not let them speak to their families. As one participant said, they are required to comply with any request the men make:

“In the forest I suffered from violence and sexual assault at the hands of a man, ... I had to say yes to anything so they would help me make the crossing in small boat.”

(WIM-22)

WIMs live in constant threat of police raids that force them to flee and disperse. If they are detained, WIMs report that the Moroccan police makes deportations without guarantees to other cities or the country border.

“In the forest, the police caught me. They left me beyond Casablanca and I walked for days to get to where the boat left.”

(WIM-1)

WIMs may also live in small apartments or flats on the cities’ outskirts, in very poor hygienic and sanitary conditions. They may remain detained in those crowded spaces for days or even years in semi-freedom while saving up money in order to pay back their debt.

“When I got to Casablanca, there was a man waiting for me who took me to a house where there were more girls. They took my passport and they forced us into prostitution, always under their close watch, abuse ... it was horrible!”

(WIM-16)

WIMs find themselves obligated to beg and hustle in order to survive and pay back their debt to the trafficking network. As one WIM says, they go into homes of Moroccans to work. They say they work as cleaners but you can sense that they are being sexually exploited:

“I moved into the house and the owner and I agreed I could do the housework and cooking. I left after a month because I was under a lot of pressure to get into prostitution.”

(WIM-12)

During the first stage of the journey, the figure of the “journey husband” arises. This is a man with whom the WIM establishes an emotional attachment. He protects the WIM and gets her pregnant. These men, chosen by the network to control the women throughout the journey, monitor their stay in Morocco, preparing them for their journey by small boat to Spain.

“I met this man, I got pregnant, I had the boy but this man disappeared. My “journey husband” organized the trip. I gave him 4000 Dirham (Moroccan currency) that I earned and he took care of everything...”

(WIM-7)

#### 3.2.3. The Dangerous Journey by Small Boat

This is an extremely dangerous journey done in small, substandard boats with hardly any water or food for the journey and surrounded by gasoline tanks and, sometimes, drugs. Without prior warning, the trafficking network puts between 35 and 60 men, women, and children on one small boat, all piled up and huddled together without space. They embark without life jackets despite the majority not knowing how to swim:

“It was a very hard journey, as the water came into the small boat. My friend’s son fell into the sea and I was able to get him out, but he wasn’t breathing … I still feel panic and anxiety from it!”

(WIM-10)

On the outskirts of the city and in the forest there are men and women who prepare and negotiate the trip. Their “journey husbands” stay in Morocco to continue their job within the human trafficking ring. The women do not know anything related to the organization, the payment, and the conditions of the journey. The small boats are very low-quality, they do not have compasses and they are not tested to see if they work prior to the journey. As one WIM says, they fit as many people as possible on the boat and they are obligated to stay still, to cover themselves with a blue tarp if they see police helicopters, and to not give up the identity of the operator of the boat:

“You get on the boat with what you have on, they don’t want you to take anything. It’s scary just to look, … they threaten us, hit us, frisk us, and rob us.”

(WIM-4)

The small boats lack space and the migrants cannot move, they suffer from headaches, dizziness, and vomiting. The small children sit on their laps the whole time, which causes them to arrive completely exhausted. As one WIM describes:

“There were 33 of us in the boat and you have to sit on the floor or on someone else’s legs. We arrived tired, in pain, with our limbs numb, and with burns.”

(WIM-7)

During the journey, WIMs experience hunger, thirst, extreme cold (they do not travel with adequate clothing), and sometimes can get sexually abused. In addition, WIMs cannot urinate and they arrive on land with excessive urine retention.

“We didn’t eat anything, were dehydrated and you can’t urinate. The men could just pull their pants down, but the women couldn’t…you soil yourself!”

(WIM-2)

### 3.3. WIM in Small Boat Should Raise the Alarm

WIMs are a very vulnerable group that requires specialized emergency care upon their arrival.

#### 3.3.1. Meeting Needs and Detecting Victims of Human Trafficking

When a small boat arrives to the Spanish coast, IMs receive emergency care. WIMs arrive exhausted from a long migratory journey full of violence, abuse, and discrimination. For our participants, being black and a WIM makes the journey especially perilous:

“There are more opportunities for them to do bad things to a woman, … I would never do this trip by small boat again.”

(WIM-5)

IMs require evaluation of their physical health, nutrition, hydration, waste elimination, injuries, burns, and wounds. In addition, WIMs also need a gynecological examination focused on the detection of sexually transmitted diseases, clandestine abortion practices, female genital mutilation, and signs of abuse. As one WIM points out:

“I arrived pregnant, they took me to the hospital and examined me, ... I was HIV positive. My daughter and I are now taking pills as treatment.”

(WIM-22)

Detecting human trafficking victims is key in the provision of emergency care. Being controlled by the trafficking network, in conjunction with feeling fearful, deters WIMs from reporting their situation and seeking protection.

“A man helped me to cross without charging me anything, I know I have a debt to pay, ... someday he’ll find me and I’ll have to pay.”

(WIM-18)

WIMs may be accompanied by a woman who ensures that they follow the orders of the network. Although during triage they remain in silence, healthcare providers can observe if WIMs lower their head upon making eye contact with another woman; the latter may be the “leader” of the group.

“My parents died and I was left in charge of my 4 siblings, and clothing sales were not doing well. Helen (the madame) urged me to come to Europe, saying, ‘There you’ll earn money, you can feed your siblings!’ She organized the journey to Morocco, but then she disappeared.”

(WIM-25)

#### 3.3.2. The Mother/Child Relationship: An Indicator of WIMs Trafficking

Some WIMs travel with their children, but when they go to board the boat, they may be separated by the organizers. As one participant said, some children may end up traveling alone, with another family member, or with strangers.

“My daughter did the journey by small boat with my niece, and they wouldn’t let me get on. When she arrived in Spain she lied and said it was her daughter, ... later that her parents were in Morocco. My daughter is now in a center for inmigrant children.”

(WIM-11)

When any minor arrives to the coast, it is important to detect the family relationship they have with the adult that accompanies them. Detecting this vulnerability is important for the safety and protection of the family, especially of women and children. As one WIM explains:

“I arrived with my little son, my other daughter had gone ahead in small boat a few weeks before. She had to travel with a stranger …”

(WIM-26)

Small boats of sub-Saharan Africans with women and children aboard need specific treatment. During triage, the organizations that provide emergency care to WIMs carry out specific protocols for detecting human trafficking, gender violence, or family separation. They carry out immediate interventions, involving the WIMs themselves.

“The Red Cross Emergency Response Team interviewed me upon arrival, the first thing I told them was that my other young daughter had arrived in Spain a few weeks prior, ... I don’t know where she is! They said they would look for her, that they would help me.”

(WIM-26)

## 4. Discussion

The objective of our study is to describe and understand the violence against WIMs who arrive in Spain in small boats. The framework developed by Zimmerman et al. [30] has allowed us to understand the experiences of WIMs in the recruitment, journey, and emergency care stages. Poverty, marginalization, and social discrimination push these women to undertake an uncertain and dangerous journey [6]. WIMs flee from a culture of exploitation and violence in their countries of origin, where they suffer abuse, arranged marriages, genital mutilation, homophobia, and discrimination [4,34]. According to Belknap [27], women migrate in search of work, education, and safety. The loss of a father/husband deprives them of safety, and pressure from their social environment triggers them to emigrate [7,35]. In the “recruitment stage” [30], the trafficking network sets the amount and the method of payment and WIMs abandon their families and start the migration process [36]. In the “travel–transit stage”, WIMs are controlled by the network and they accumulate a debt that is difficult to pay back [37]. In line with our own results [22,38], WIMs become aware as they are raped or prostituted that they are paying for the journey to cross the border. According to other studies [39], dependence on human smugglers and vulnerability to traffickers leads to the systematization and normalization of sexual violence en route. WIMs are also subject to strict reproductive control [18], if they become pregnant, the human trafficking ring may give them medicines that provoke clandestine abortions in high-risk sanitary conditions [35]. According to Keygnaert et al. [40], WIMs are kept in overcrowded spaces where they suffer social and sexual exploitation. Prior to boarding, WIMs wait in camps, where they are cut off from all communication and with restricted freedom [35]. In the “travel–transit stage”, WIMs cross the Mediterranean in small overcrowded boats; they are unable to move during the entire dangerous journey [41]. Our study concurs with Trovato et al. [6] in that food and water are scarce, violence is common, and WIMs also can suffer from sexual abuse during their journey [42]. In the detention stage [30], WIMs feel exhausted and uncertain of what their future holds. WIMs need emergency care that meets their physical, psychological, and spiritual needs [43,44]. According to Women’s Link Worldwide [35], our results suggest looking for signs of coercion, presence of “madames”, violence, and human trafficking, getting them to break their silence and allowing them to communicate with friends and family. Taking a multidisciplinary approach is a key element in the protection of WIMs [45]. Although it is the police’s job to identify victims of human trafficking, healthcare providers can help through gynecological examinations or pregnancy tests [29], looking for signs of infection, tearing, and female mutilation [21,24]. As in other studies [45], our results raise the point that healthcare providers and police could work together to look for signs of violence against and human trafficking of WIMs who arrive in small boats. According to Keygnaert et al. [40], healthcare providers can ask about sexual abuse, about whether the women have a partner, or about the identity of the father of the baby. Although WIMs may travel with their children, the trafficking network can force women to be separated from them or to take children that are not theirs [46,47]. Our results also show that monitoring the WIM–child relationship is one of the key ways of detecting human trafficking, keeping in mind the country of origin of each WIM [26]. Violence against WIMs is widely recognized as gender-based violence, and states are obligated to act by taking a comprehensive approach [28]. As in other studies [27,48], ours found that healthcare providers could improve the care given to WIMs as well as detect the violence against them by better comprehending their needs and developing specifically adapted programs to meet them. This process should have a later follow-up due to the violence WIMs may continue to experience during the exploitation and reintegration stages [12].

### Limitations

Several women refused to participate in the study out of fear of speaking out or retaliation from human trafficking networks. The sample of WIMs is nonhomogenous, as their economic, political, and sociocultural characteristics vary according to their country of origin.

## 5. Conclusions

Many WIMs live in a culture of oppression, discrimination, and inequality that drives them to emigrate to Europe from their home countries in search of a better life. Although they are familiar with the risks and encouraged by false promises, they are still recruited by the trafficking network to migrate to Europe. During this dangerous journey, they suffer from hunger, dehydration, or injuries. Additionally, they also incur a huge debt that exposes them to sexual abuse and rape. WIMs are abandoned in Morocco or Algeria; they live in conditions of semi-slavery and prostitution and their bodies act as currency to be traded with local people and police. The trafficking network controls their sexual and reproductive rights, their pregnancy and their abortions. After a long wait, WIMs cross the Mediterranean Sea in small boats. Upon their arrival in Spain, WIMs require emergency care with a special focus on nutrition, hydration, hypothermia, and injuries. In addition, healthcare providers must provide gynecological examinations, detect human trafficking victims and guarantee their safety. The development of joint protocols between governments, NGOs, and police and healthcare providers, along with the observation of the relationship between WIMs and children are crucial steps to improving emergency care. These results may serve to better administer care to WIMs who reach Europe by sea from the north of Africa.

## Figures and Tables

**Table 1:** Sociodemographic data of the participants (*N* = 26).

Participant	Sex	Age	Port of Arrival	Travel Status	Country
WIM-1	Female	29	Almería	Pregnant	Nigeria
WIM-2	Female	34	Almería	Son (9 years old), Pregnant	Algeria
WIM-3	Female	19	Almería	Alone	Gambia
WIM-4	Female	18	Almería	Brother (6 years old)	Guinea
WIM-5	Female	33	Almeria	Pregnant	Ivory Coast
WIM-6	Female	34	Almería	Alone	Nigeria
WIM-7	Female	35	Cadiz	Son (2 years old), Pregnant	Morocco
WIM-8	Female	29	Motril (Granada)	Alone	Senegal
WIM-9	Female	23	Almería	Alone	Gambia
WIM-10	Female	35	Almería	Daughter (10 years old)	Ivory Coast
WIM-11	Female	35	Cadiz	Daughter (3 years old)	Ivory Coast
WIM-12	Female	21	Almería	Son (4 years old)	Cameroon
WIM-13	Female	39	Motril (Granada)	Pregnant	Ivory Coast
WIM-14	Female	29	Almería	Alone	Sierra Leona
WIM-15	Female	27	Almería	Daughter (4 years old)	Guinea
WIM-16	Female	28	Tarifa (Cádiz)	Alone	Nigeria
WIM-17	Female	27	Barbate (Cádiz)	Alone	Ivory Coast
WIM-18	Female	24	Almería	Pregnant	Guinea
WIM-19	Female	42	Almería	Alone	Guinea
WIM-20	Female	29	Motril (Granada)	Alone	Guinea
WIM-21	Female	29	Almería	Pregnant	Cameroon
WIM-22	Female	20	Almería	Pregnant	Ivory Coast
WIM-23	Female	29	Ceuta	Alone	Guinea
WIM-24	Female	35	Almería	Daughter (1 año)	Ivory Coast
WIM-25	Female	24	Almería	Alone	Nigeria
WIM-26	Female	24	Almería	Son (1 año)	Guinea

WIM = women irregular migrant.

**Table 2:** Interview protocol.

Stage	Subject	Content/Example Questions
Introduction	Motives, reasons	Understand experiences of violence towards WIMs during their migratory journey to Spain.
	Ethics issues	Ask them about their willingness to take part, inform them about the recording, consent, possibility of dropping out, confidentiality.
Beginning	Introductory question	What was your experience regarding violence on the migratory journey?
Development	Conversation guide	Can you tell me about your home country, your family life and your work history?
		When and why did you decide to leave your country? How did you arrange the journey?
		Could you tell me about your migratory journey?
		Tell us about any violent experiences and situations you have lived through on your way to coming to Spain in a small boat.
		What is your opinion about the difficulty of undergoing the migratory journey, being a woman, pregnant or traveling with a child?
Closing	Final question	Is there anything else you would like to tell me?
	Appreciation	Thank them for taking part, remind them that their testimony will be of great use, and place ourselves at their disposition.

WMIs = women irregular migrants.

**Table 3:** Themes, subthemes, and units of meaning.

Theme	Subtheme	Units of Meaning
Poverty and discrimination push WIMs into migrating.	The difficulty of living in social and economic inequality.	Poverty, social marginalization, death or abandonment of the father/husband, forced marriage.
	Escaping a culture of discrimination against women.	Feminine genital mutilation, domestic abuse, traditions that invalidate women.
	Recruiting WIMs in their countries of origin.	Recruiters, scams, coercive magic, managing debts, organizing the trip, kidnapping.
WIMs as a paradigm of extreme vulnerability.	Sexual and reproductive control: the journey husband.	Migratory routes, control, threats, avoiding frontiers (fence), scams, promises, debts, lack of reproductive choice, accompanied, children.
	Waiting to board: between worker exploitation and sexual slavery.	Abuse by the local population, police raids, “small boat flats”, detained and exploited, life in the forest, cut off from communication, journey husband.
	The dangerous journey by small boat.	Lack of security, ignorance, uncertainty, without moving, plundered, law of silence, elimination, food, clothing, sexual abuse.
WIM in small boat should raise the alarm.	Meeting needs and detecting victims of human trafficking.	Trip without guarantees, physical injuries, threats, fear, silence, gynecological examination, pregnant, alone, victims of work and sexual exploitation, the “madame”
	The mother/child relationship: an indicator of WIMs trafficking.	Human trafficking, awareness of the problem, active observation, bond.

WIM = women irregular migrant.
